# Unsteady hybrid nanofluid ($UO_2$, MWCNTs/blood) flow between two rotating stretchable disks with chemical reaction and activation energy under the influence of convective boundaries

**DOI:** 10.1038/s41598-023-32606-4

**Published:** 2023-04-15

**Authors:** Mubashir Qayyum, Sidra Afzal, Mohamed R. Ali, Muhammad Sohail, Naveed Imran, Gilbert Chambashi

**Affiliations:** 1 National University of Computer and Emerging Sciences FAST Lahore, Lahore, Pakistan; 2 Faculty of Engineering and Technology, Future University in Egypt New Cairo, 11835 Cairo, Egypt; 3 Institute of Mathematics, Khwaja Fareed University of Engineering and Information Technology, Rahim Yar Khan, 64200 Pakistan; 4 HITEC Colleges, HIT Taxila Cantt, Taxila, Pakistan; 5 School of Business Studies, Unicaf University, Longacres, Lusaka, Zambia

**Keywords:** Mathematics and computing, Nanoscience and technology

## Abstract

Hybrid nanofluids are extensively analyzed in recent studies due to their better performance in numerous areas such as heat and mass transfer enhancement, biological fluid movement, medical equipment, heat exchangers, electronic cooling and automotive industry. In current study the nanoparticle concentration utilized is much important in biomedical industry. Major applications include drug delivery, radio-pharmaceuticals, centrifuging blood to obtain red blood cells and plasma, medical implants, onco therapeutics and photo thermal cancer therapy. In this regard, the primary focus of this study is to simulate a blood based unsteady hybrid nanofluid flow between two rotating, stretching disks and convective boundaries. The two nanoparticles in this study are uranium dioxide $UO_{2}$ and multi-walled carbon nanotubes *MWCNTs*. The hybrid nanofluid is under the influence of magnetohydrodynamic effects and chemical reaction with activation energy. The governing partial differential equations (PDEs) are transformed into ordinary differential equations (ODEs) using suitable similarity transform. Homotopy analysis method is used to solve the non-linear system of ODEs and $\hbar$-curves are plotted to find suitable region of $\hbar _{i}$ for convergent series solution. Velocity profile is examined for axial, radial and tangential direction against various fluid parameters. Temperature and concentration profiles are analyzed for both convective and non-convective cases. It is observed that convective boundaries result in elevated temperature when compared with non-convective case. Moreover, skin friction, heat and mass transfer rates are also examined with respect to changing volume fraction $\varphi _{UO_{2}}$.The results revealed that skin friction and rate of heat transfer increases with increase in volume fraction of both nanoparticles $UO_{2}$ and *MWCNTs* while the mass transfer rate depicts contrasting behavior.

## Introduction

The nanofluids are formed by adding particles of nano-meter size into base fluid either by directly mixing (one-step method) or synthesizing nanoparticles first and then mixing (two-step method). The nanofluids consist of two-phases, fluid phase (base-fluid) and solid-phase (nanoparticles). Base fluids usually are water, ethylene glycol, blood and engine oil etc. while nanoparticles are mostly metal oxides, carbides or carbon nanotubes CNTs. When two types of nanoparticles are mixed into the base fluid then hybrid nanofluid is formed. Hybrid nanofluids enhance the efficiency of base fluids in terms of effective thermal conductivity, diffusivity, viscosity and heat transfer rates. These fluids are potentially useful in microelectronics, hybrid-powered engines, solar energy collectors, heat exchangers, drug transport and many medical equipment. Due to vast applicability, many researchers have attempted to analyze and simulate various hybrid nanofluid models in most recent studies. Rashidi et al.^1^ simulated the flow of a nanofluid over a porous rotating disk impacted by magnetohydrodynamic forces. Jabbaripour et al.^2^ examined a water based three dimensional hybrid nanofluid flow with aluminum and copper nanoparticles at slip boundary conditions. Subhani and Nadeem^3^ analyzed water based micro-polar hybrid nanofluid flow with copper and titanium oxide nanoparticles over a stretching surface. Khan et al.^4^ studied the convective flow of Casson-nanofluid with gold nanoparticles through a rotating disk under impact of non-linear thermal radiation. Izaday et al.^5^ numerically scrutinized a $CuO{-}Fe_{2}O_{3}$/water based hybrid nanofluid through Tiwari-Das model. Blood hybrid nanofluid with $TiO_{2}$ and *Ag* nanoparticles passing through an artery is analyzed by Chahregh and Dianrvand^6^ to better understand the blood circulation through the respiratory system. Ghasemian et al.^7^ presented their work on three-dimensional unsteady Maxwell nanofluid. Alghamdi et al.^8^ examined the flow of a $Cu+CuO$/blood hybrid nanofluid through two permeable channels with magnetohydrodynamic effects. Dinarvand et al.^9^ conducted a study on flow of $Cu+CuO$/blood hybrid nanofluid past a porous stretching sheet near a stagnation point with special reference to drug transport. Sheikholeslami et al.^10^ considered MFD viscosity effects on flow of a magnetic nanofluid. Waqas et al.^11^ simulated the free convective flow of a water based nanofluid. $SWCNTs-TiO_{2}/MWCNTs+CoFe_{2}O_{4}$ nanoparticles over a single rotating disk with MHD effect. Dinarvand and Nejad^12^ simulated a stagnation point flow of a hybrid nanofluid with respect to the masses of two types of nanoparticles i.e., magnesium oxide and silver. Mansourian et al.^13^ analyzed the flow of a ferro-hybrid nanofluid passing over a stretching sheet.

Flow originated by rotating stretching disks have gathered researchers’ interest due to their applications in food processing, medical equipment, industrial and engineering sectors. Fruitful outcomes have been drawn through several researches but the pioneering work on rotating disk was presented by Karman^14^. He developed a differential setup to analyze the hydrodynamical flow over an infinite rotating disk. His work was further extended by Griffiths^15^ where he analyzed generalized Newtonian fluids which provided a comprehensive description to non-Newtonian boundary layer flows. Rashidi et al.^16^ investigated the slip flow of a nanofluid on a rotating porous disk. Khan et al.^17^ numerically analyzed Oldroyd-B nanofluid flowing over an exponentially stretched surface with radiative effects. Hayat et al.^18^ studied a third grade nanofluid flow over a single rotating and stretching disk with thermophoresis and Brownian motion. Shah et al.^19^ investigated a nanofluid flow between two rotating and stretching disks with silver based CNTs under MHD effects. Usman et al.^20^ presented work on enhancement of heat transfer in a blood based nanofluid with power-law model and heat source/sink stimulated by two rotating stretchable disks. Convective flow of a Newtonian fluid between co-rotating stretching disks with Soret and Dufour effects is examined by Sharma et al.^21^. Rauf et al.^22^ explored the rate of heat transfer in a hybrid ferrofluid boundary layer flow passing over a rotating and non-linearly stretching disk in presence of an alternating magnetic field. Usman et al.^23^ presented steady flow model of a power law fluid co-axially rotating between two stretchable disks with heat source/sink.

Addition of surfactants, stabilizers and various nanoparticles in blood causes major chemical reactions. Activation energy is the minimum amount of energy required to start off that chemical reaction. Arrhenius equation is utilized to describe the change of rate constant with changing temperature in a chemical reaction. These reactions take place mostly in chemical reactors which are most of the time limited through the rate of mass transfer. In this context, it becomes much important to incorporate chemical reaction effects in order to analyze the flow problem in this study. Other researchers including Hamid et al.^24^, Salahuddin et al.^25^, McCash et al.^26^, Raza et al.^27^ and Nisar et al.^28^ explored effects of chemical reaction with activation energy on various non-Newtonian and nanofluid models. Saleem et al.^29^ studied the effects of chemical reaction on flow of a second-order viscoelastic fluid with heat generation effect. Gowda et al.^30^ recently analyzed the heat and mass transfer rate in a non-Newtonian second grade nanofluid model undergoing chemical reaction with activation energy. Zaib et al.^31^ applied binary chemical reaction with MHD effects on Casson nanofluid flowing over a wedge. Khan et al.^32^ analyzed a chemically reactive nanofluid flow over a moving needle with viscous dissipation. Flow of cross nanofluid with immersed gyrotatic microorganisms is presented by Azam et al.^33^ under effects of non-linear thermal radiation.

Convective conditions are characterized by interaction between boundaries of the machinery and the surrounding environment. Heat exchange by convection is the main cause of thermal response in the machine tools which is important in describing the fluctuations due to environmental changes or addition of cooling liquid. The heat transfer coefficients (HTCs) in these conditions are the proportionality constants when convective heat flux and temperature difference (between fluid and structure) are related. Aziz^34^ in 2009 pioneered working with convective boundary conditions while investigating the Blasius flow. Afterwards, various authors analyzed different fluid models in this regard. Yao et al.^35^ analyzed the heat transfer in viscous fluid flow past a stretching/shrinking wall with a convective boundary condition. Hayat et al.^36^ investigated the stagnation point flow of a Casson fluid with mixed convection over a linearly stretching surface with thermally convective boundary. Wang et al.^37^ studied the bio-convective flow of a Maxwell nanofluid with slip effects and passing over an exponentially stretched surface. Haq et al.^38^ investigated the flow behavior of a Casson nanofluid with convective boundaries. Zaib et al.^39^ simulated flow of a nanofluid with convective boundaries in a Darcy-Brinkman porous medium. Boundary layer flow of a Casson fluid is examined by Hussain et al.^40^ with convective boundary conditions and flow over a stretching wedge. Furthermore, recent researchers on convective boundaries are presented by various authors including Akhtar et al.^41^, Anuar et al.^42^, Mabood et al.^43^, Becerro et al.^44^ and Rasheed et al.^45^.

In light of the literature review stated above, it is observed that an unsteady flow of a blood base hybrid nanofluid between two rotating and stretching disks with chemical reaction and activation energy along with convective boundaries has not been investigated. Moreover, uniqueness and need of present work is discussed in comparison with existing recent literature on unsteady hybrid nanofluid flow in Table 1. In this regard, current study focuses on the analysis of a blood based hybrid nanofluid under aforementioned phenomena. The problem in discussion finds its major applicability in rotating disk blood oxygenators and centrifuge machinery to separate red-blood cells, plasma and platelets from human blood. The nanoparticles considered in this case are uranium dioxide $UO_{2}$ and multi-walled carbon nanotubes *MWCNTs*. The governing unsteady non-linear PDEs are converted into ODEs by applying suitable similarity transformations. The obtained system of ODEs is then solved by using homotopy analysis method (HAM)^46^. To provide convergence of the solution $\hbar$-curves are plotted and convergent series solutions are tabulated at various order of approximations. Graphical analysis on velocity, temperature and concentration profile is physically interpreted. Furthermore, skin friction, heat and mass transfer rates are also simulated against increasing volume fractions $\varphi _{i}$ of both nanoparticles. This provides useful numerical results that depict behavior of hybrid nanofluid in both convective and non-convective boundary cases.

**Table 1:** Comparison of present study with recent work on unsteady blood based hybrid nanofluid.

	Unsteady flow	Stretch disks	Rotating disks	Convec. boundary	Chem. reac.	Heat transfer	Mass transfer
Kashi’ie et al.^47^	Yes	Yes	No	No	No	Yes	No
Rehman and Salleh^48^	Yes	Yes	No	No	No	Yes	No
Gandhi and Sharma^49^	Yes	No	No	No	No	Yes	No
Present study	Yes	Yes	Yes	Yes	Yes	Yes	Yes

## Mathematical formulation

**Figure 1:**
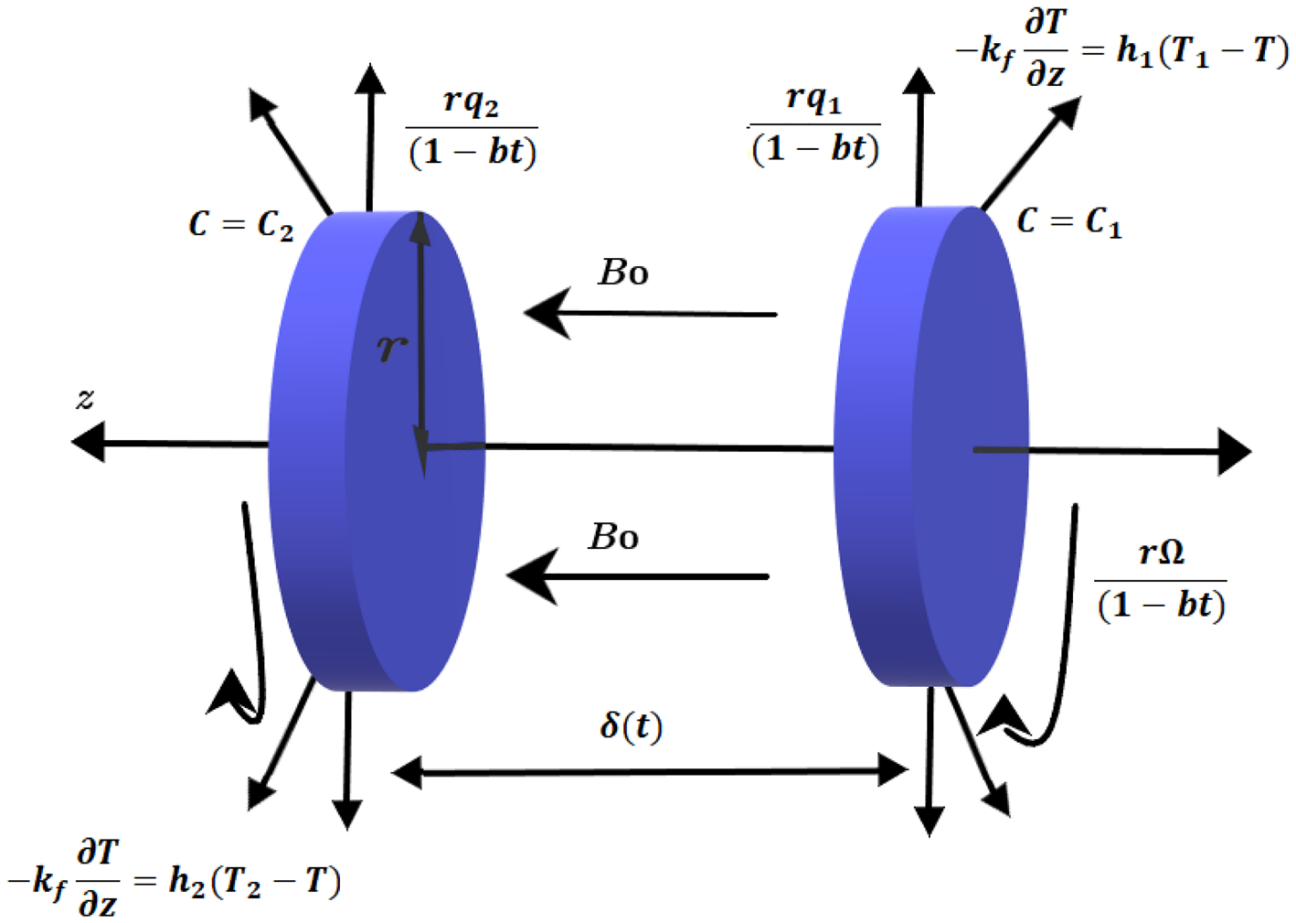
Blood flow geometry.

We consider an unsteady blood hybrid nanofluid flow between two rotating and stretching disks in cylindrical coordinate system $(r,\phi , z)$. The stretching rate of right disk is $q_{1}$ and left disk is $q_{2}$. Both disks rotate with frequency $\Omega$. Temperature and concentration at right and left disk is $T_{1},C_{1}$ and $T_{2},C_{2}$, respectively. A constant magnetic field of intensity $B_{0}$ is applied axially between two disks that are $\delta (t)$ apart. The hybrid nanofluid is impacted by magnetohydrodynamic effects and chemical reaction with activation energy. Complete flow profile of the problem is depicted in Fig. 1. The continuity, momentum, temperature and concentration equations are as follows

1 $$\begin{aligned}{} & {} \frac{\partial u}{\partial r}+\frac{\partial w}{\partial z}+\frac{u}{r}= 0, \end{aligned}$$

2 $$\begin{aligned}{} & {} \frac{\partial u}{\partial t}+u\frac{\partial u}{\partial r}+w\frac{\partial u}{\partial z}-\nu _{hnf}\nabla ^{2}u-\frac{v^{2}}{r}+\frac{\sigma _{hnf}B^{2}_{0}}{\rho _{hnf}}u=0, \end{aligned}$$

3 $$\begin{aligned}{} & {} \frac{\partial v}{\partial t}+u\frac{\partial v}{\partial r}+w\frac{\partial v}{\partial z} -\nu _{hnf}\nabla ^{2}v+\frac{uv}{r}+\frac{\sigma _{hnf}B^{2}_{0}}{\rho _{hnf}}v=0, \end{aligned}$$

4 $$\begin{aligned}{} & {} \frac{\partial T}{\partial t}+u\frac{\partial T}{\partial r}+w\frac{\partial T}{\partial z} = \frac{k_{hnf}}{(\rho Cp)_{hnf}}\nabla ^{2}T, \end{aligned}$$

5 $$\begin{aligned}{} & {} \frac{\partial C}{\partial t}+u\frac{\partial C}{\partial r}+w\frac{\partial C}{\partial z} =D_{hnf} \frac{\partial ^{2} C}{\partial z^{2}}-{\mathbb {K}}_{r}^{2}(C-C_{2})\left( \frac{T}{T_{2}}\right) ^{s} e^{\frac{-{\mathfrak {E}}}{{\mathfrak {K}}T}}, \end{aligned}$$

subject to the following boundary conditions

6 $$\begin{aligned} \begin{aligned} u&=\frac{q_{1}r}{1-bt},\quad v=\frac{r \Omega }{1-bt},\quad w=0\quad -k_{f}\frac{\partial T}{\partial z}={\mathfrak {h}}_{1} (T_{1}-T),\quad C=C_{1}\quad at\quad z=0\\ u&=\frac{q_{2}r}{1-bt},\quad v=0,\quad -k_{f}\frac{\partial T}{\partial z}={\mathfrak {h}}_{2} (T_{2}-T),\quad C=C_{2}\quad at\quad z=\delta (t)=\sqrt{\frac{\nu }{\Omega }(1-bt)} \end{aligned} \end{aligned}$$

here *u*, *v* and *w* are radial, tangential and axial velocities in *r*, $\phi$ and *z* directions, respectively. *T* represents hybrid nanofluid temperature and *C* is the concentration. Chemical reaction rate is denoted by ${\mathbb {K}}_{r}$, energy to start the chemical reaction is ${\mathfrak {E}}$ and *s* is a constant power. Also, $1-bt>0$ and *b* is a positive constant with dimension of $(time)^{-1}$. $\nu$ is the kinematic viscosity, $\sigma$ is the electrical conductivity, *k* is thermal conductivity and *D* is the thermal diffusivity. ${\mathfrak {h}}_{1}$ and ${\mathfrak {h}}_{2}$ are the heat transfer coefficients at right and left disks, respectively. The subscript $'hnf'$ corresponds to the hybrid nanofluid quantity and $'f'$ corresponds to the base fluid quantity which are defined in Table 2. The thermophysical properties of base fluid, blood and two nanoparticles uranium dioxide $UO_{2}$ and multi-walled carbon nanotubes *MWCNTs* are presented in Table 3.

**Table 2:** Maxwell model for thermophysical characteristics of the hybrid nanofluid^50–53^

Properties	Hybrid nanofluid
Kinematic viscosity	$\nu _{hnf}=\frac{\mu _{hnf}}{\rho _{hnf}}$
Thermal diffusivity	$\frac{D_{hnf}}{D_{f}}=(1-\varphi _{hnf})$
Volume fraction	$\varphi _{hnf}=\varphi _{UO_{2}}+\varphi _{CNTs}$
Dynamic viscosity	$\mu _{hnf}=\frac{\mu _{f}}{(1-\varphi _{CNTs})^{5/2}(1-\varphi _{UO_{2}})^{5/2}}$
Density	$\rho _{hnf}=(1-\varphi _{hnf})\rho _{f}+\varphi _{CNTs}\rho _{CNTs}+\varphi _{UO_{2}}\rho _{UO_{2}}$
Thermal conductivity	$\frac{k_{hnf}}{k_{f}}=\frac{\varphi _{k}+2k_{f}+2(\varphi _{CNTs}k_{CNTs}+\varphi _{UO_{2}}k_{UO_{2}})-2\varphi _{hnf}k_{f}}{\varphi _{k}+2k_{f}-(\varphi _{CNTs}k_{CNTs}+\varphi _{UO_{2}}k_{UO_{2}})+\varphi _{hnf}k_{f}}$
	where $\varphi _{k}=\frac{\varphi _{CNTs}k_{CNTs}+\varphi _{UO_{2}}k_{UO_{2}}}{\varphi _{hnf}}$
Electrical conductivity	$\frac{\sigma _{hnf}}{\sigma _{f}}=\frac{\varphi _{\sigma }+2k_{f}+2(\varphi _{CNTs}\sigma _{CNTs}+\varphi _{UO_{2}}\sigma _{UO_{2}})-2\varphi _{hnf}\sigma _{f}}{\varphi _{\sigma }+2k_{f}-(\varphi _{CNTs}\sigma _{CNTs}+\varphi _{UO_{2}}\sigma _{UO_{2}})+\varphi _{hnf}\sigma _{f}}$
	where $\varphi _{\sigma }=\frac{\varphi _{CNTs}\sigma _{CNTs}+\varphi _{UO_{2}}\sigma _{UO_{2}}}{\varphi _{hnf}}$
Heat capacity	$(\rho Cp)_{hnf}=(1-\varphi _{hnf})(\rho Cp)_{f}+\varphi _{CNTs}(\rho Cp)_{CNTs}+\varphi _{UO_{2}}(\rho Cp)_{UO_{2}}$

**Table 3:** Thermophysical properties of blood, $UO_{2}$ and *MWCNTs*^6,54,55^.

Physical properties	Blood	$UO_{2}$	*MWCNTs*
$\rho (\mathrm{kg/m}^{3})$	1053	10970	1600
$C_{p}$ (J/gK)	3594	235	796
k(W/mK)	0.492	8.68	3000
$\sigma$ (S/m)	0.8	0.029	$1.9\times 10^{-4}$

In order to non-dimensionalize the problem and to convert the system of partial differential equations into ordinary ones , the following similarity transforms are introduced^56,57^

7 $$\begin{aligned} \begin{aligned} u=\frac{\Omega r}{1-bt} F'(\eta ),\quad v=\frac{\Omega r}{1-bt}G(\eta ),\quad w=-2\sqrt{\frac{\Omega \nu _{f}}{1-bt}}F(\eta ),\\ \eta =z\sqrt{\frac{\Omega }{\nu _{f}(1-bt)}},\quad \theta (\eta )=\frac{T-T_{2}}{T_{1}-T_{2}},\quad \phi (\eta )=\frac{C-C_{2}}{C_{1}-C_{2}}\\ \end{aligned} \end{aligned}$$

We use Eq. (7) in Eqs. (1)–(4) and obtain following system of non-dimensional ordinary differential equations for the flow problem

8 $$\begin{aligned} \varsigma _{3}F'''-F'^{2}+2FF''+\frac{\varsigma _{1}}{\varsigma _{2}}MF'-{\mathbb {U}}\left( F'+\frac{\eta }{2}F''\right) -G^{2}=0, \end{aligned}$$

9 $$\begin{aligned} \varsigma _{2}\varsigma _{3}G''-\varsigma _{2}{\mathbb {U}}\left( G+\frac{\eta }{2}G'\right) -\varsigma _{1}MG-2\varsigma _{2}(GF'-FG')=0, \end{aligned}$$

10 $$\begin{aligned} \varsigma _{4}\theta ''-Pr\varsigma _{5}({\mathbb {U}}\eta -2F)\theta '=0, \end{aligned}$$

11 $$\begin{aligned} \varsigma _{6}\phi ''-{\mathcal {S}}_{c}\left( {\mathbb {U}}\eta +\kappa _{t}(1+\gamma _{1}\theta )^{s}\exp \left[ \frac{-{\mathfrak {E}}_{t}}{1+\gamma _{1}\theta }\right] \right) \phi +2{\mathcal {S}}_{c}F\phi '=0, \end{aligned}$$

with boundary conditions as follows

12 $$\begin{aligned} \begin{aligned}{}&F'(0)=\alpha _{1},\quad F(0)=0,\quad G(0)=1,\quad \theta '(0)=-{\mathfrak {B}}_{i1}\{1-\theta (0)\},\quad \phi (0)=1,\quad at \quad \eta =0, \\&F'(1)=\alpha _{2},\quad G(1)=0,\quad F(1)=0,\quad \theta '(1)={\mathfrak {B}}_{i2}\theta (1),\quad \phi (1)=0,\quad at\quad \eta =1. \end{aligned} \end{aligned}$$

In Eqs. (8)–(12) the dimensionless quantities are

13 $$\begin{aligned} \begin{aligned}{}&\varsigma _{1}=\frac{\sigma _{hnf}}{\sigma _{f}},\quad \varsigma _{2}=\frac{\rho _{hnf}}{\rho _{f}},\quad \varsigma _{3}=\frac{\nu _{hnf}}{\nu _{f}},\quad \varsigma _{4}=\frac{k_{hnf}}{k_{f}},\quad \varsigma _{5}=\frac{(\rho Cp)_{hnf}}{\rho Cp_{f}}, \\&\varsigma _{6}=\frac{D_{hnf}}{D_{f}},\quad {\mathbb {U}}=\frac{b}{\Omega },\quad Pr=\frac{(\rho Cp)_{f}\nu _{f}}{k_{f}},\quad T_{w}=\frac{T_{1}}{T_{2}}\quad \alpha _{1}=\frac{q_{1}}{\Omega }, \\&\kappa _{t}=\frac{{\mathbb {K}}_{r}^{2}(1-bt)}{\Omega },\quad {\mathcal {S}}_{c}=\frac{\nu _{f}}{D_{f}},\quad {\mathbb {E}}_{t}=\frac{-{\mathfrak {E}}}{{\mathfrak {K}}T_{2}},\quad {\mathfrak {B}}_{i1}=\frac{{\mathfrak {h}}_{1}}{k_{f}}\sqrt{\frac{\nu _{f}(1-bt)}{\Omega }},\\&M=B_{0}^{2}\frac{\sigma _{f}}{\rho _{f}\Omega },\quad \alpha _{2}=\frac{q_{2}}{\Omega },\quad {\mathfrak {B}}_{i2}=\frac{{\mathfrak {h}}_{2}}{k_{f}}\sqrt{\frac{\nu _{f}(1-bt)}{\Omega }},\quad \gamma _{1}=T_{w}-1. \end{aligned} \end{aligned}$$

here $\varsigma _{i}$ are the hybrid nanofluid parameters, ${\mathbb {U}}$ is the unsteadiness parameter, *Pr* is the Prandtl number, $T_{w}$ is the temperature ratio, $\alpha _{1}$ and $\alpha _{2}$ are the stretching parameters, $\kappa _{t}$ is the chemical reaction parameter, ${\mathcal {S}}_{c}$ is the Schmidt number, ${\mathbb {E}}_{t}$ is the activation energy parameter, ${\mathfrak {B}}_{1}$ and ${\mathfrak {B}}_{2}$ are the Biot numbers and *M* is the magnetic interaction parameter.

### Skin friction, Nusselt number and Sherwood number

Skin friction ${\mathbb {C}}_{f}$ along the wall of disk, heat transfer $\mathbb{N}\mathbbm{u}$ and mass transfer $\mathbb{S}\mathbbm{h}$ are defined as

14 $$\begin{aligned} \begin{aligned} {\mathbb {C}}_{f}=\frac{\sqrt{\psi _{wr}^{2}+\psi _{w\phi }^{2}}}{\rho _{f}(\Omega r)^{2}},\quad \mathbb{N}\mathbbm{u}=\frac{r\vartheta _{r}}{k_{f}(T_{1}-T_{2})},\quad \mathbb{S}\mathbbm{h}=\frac{r{\mathfrak {Q}}_{m}}{D_{f}(C_{1}-C_{2})}, \end{aligned} \end{aligned}$$

here radial and transversal shear stress at disk $\psi _{wr},\psi _{w\phi }$, heat flux at surface $\vartheta _{r}$ and mass transfer ${\mathfrak {Q}}_{m}$ are given below

15 $$\begin{aligned} \begin{aligned}{}&\psi _{wr}=[\mu _{hnf}(u_{z}+u_{\phi })]_{z=0},\quad \psi _{w\phi }=\left[ \mu _{hnf}\left( v_{z}+\frac{1}{r}+w_{\phi }\right) \right] _{z=0},\\&\vartheta _{r}=-k_{f}(T_{z})_{z=0},\quad {\mathfrak {Q}}_{m}=-D_{hnf} \left[ \frac{\partial \phi }{\partial z}\right] _{z=0}, \end{aligned} \end{aligned}$$

By using Eq. (15) in Eq. (14) and employing the similarity transforms from Eq. (7) we obtain following non-dimensional form

16 $$\begin{aligned} \begin{aligned} Re^{-1/2}{\mathbb {C}}_{f}=\frac{\sqrt{F''(0)^{2}+G'(0)^{2}}}{(1-\varphi _{1})^{2.5}(1-\varphi _{2})^{2.5}},\quad Re^{1/2}\mathbb{N}\mathbbm{u}=-\varsigma _{4}\theta '(0),\quad Re^{1/2}Sh=-\varsigma _{6}\phi '(0). \end{aligned} \end{aligned}$$

## Solution methodology

In order to solve system of highly non-linear ordinary differential equations in Eqs. (8)–(11) we use a technique named homotopy analysis method. This is a semi-analytical approach which is quite helpful in solving non-linear ordinary and partial differential equations efficiently. Homotopy analysis method has major advantages over other analytical approaches as it provides great flexibility in the expression of series form solution in terms of various base functions. Moreover, the auxiliary parameter $\hbar$ provides a region of convergence and rate of the series solution. For solution purpose, we first develop a homotopy on the system of equations and write zeroth order deformation equation as

17 $$\begin{aligned} \begin{aligned}{}&{\mathfrak {L}}_{F}(1-\ddot{p})[F(\eta ,\ddot{p})-F_{0}(\eta )]-\ddot{p}\hbar _{F}H_{F}{\mathfrak {N}}_{F}[F(\eta ,\ddot{p}),G(\eta ,\ddot{p})]=0,\\&{\mathfrak {L}}_{G}(1-\ddot{p})[G(\eta ,\ddot{p})-G_{0}(\eta )]-\ddot{p}\hbar _{G}H_{G}{\mathfrak {N}}_{G}[G(\eta ,\ddot{p}),F(\eta ,\ddot{p})]=0,\\&{\mathfrak {L}}_{\theta }(1-\ddot{p})[\theta (\eta ,\ddot{p})-\theta _{0}(\eta )]-\ddot{p}\hbar _{\theta }H_{\theta }{\mathfrak {N}}_{\theta }[\theta (\eta ,\ddot{p}),F(\eta ,\ddot{p})]=0,\\&{\mathfrak {L}}_{\phi }(1-\ddot{p})[\phi (\eta ,\ddot{p})-\phi _{0}(\eta )]-\ddot{p}\hbar _{\phi }H_{\phi }{\mathfrak {N}}_{\phi }[\phi (\eta ,\ddot{p}),\theta (\eta ,\ddot{p})]=0, \end{aligned} \end{aligned}$$

subject to following boundary conditions

18 $$\begin{aligned} \begin{aligned}{}&F'(0,\ddot{p})=\alpha _{1},\quad F(0,\ddot{p})=0,\quad G(0,\ddot{p})=1,\quad \theta '(0,\ddot{p})=-{\mathfrak {B}}_{i1}(1-\theta (0,\ddot{p})),\quad \phi (0,\ddot{p})=1,\\&F'(1,\ddot{p})=\alpha _{2},\quad G(1,\ddot{p})=0,\quad F(1,\ddot{p})=0,\quad \theta '(1,\ddot{p})={\mathfrak {B}}_{i2}\theta (1,\ddot{p}),\quad \phi (1,\ddot{p})=0, \end{aligned} \end{aligned}$$

here ${\mathfrak {N}}_{i}$ are the non-linear operators, $\ddot{p}$ is the embedding parameter such that $\ddot{p}\in [0,1]$, also ${\mathfrak {L}}_{i}$ are the linear operators and $F_{0},G_{0},\theta _{0},\phi _{0}$ are the linear operators and initial guess which are defined below for this flow problem

19 $$\begin{aligned} \begin{aligned}{}&{\mathfrak {L}}_{F}=F_{\eta ,\eta ,\eta },\quad {\mathfrak {L}}_{G}=G_{\eta ,\eta },\quad {\mathfrak {L}}_{\theta }=\theta _{\eta ,\eta },\quad {\mathfrak {L}}_{\phi }=\phi _{\eta ,\eta }\\&F_{0}(\eta )=\alpha _{1} \eta +\frac{\alpha _{2}-\alpha _{1}}{2}\eta ^{2},\quad G_{0}(\eta )=1-\eta ,\\&\theta _{0}(\eta )=\frac{-{\mathfrak {B}}_{i1}+ {\mathfrak {B}}_{i1}{\mathfrak {B}}_{i2}-{\mathfrak {B}}_{i1}{\mathfrak {B}}_{i2}\eta }{-{\mathfrak {B}}_{i1}+{\mathfrak {B}}_{i2}+{\mathfrak {B}}_{i1}{\mathfrak {B}}_{i2}},\quad \phi _{0}(\eta )=1-\eta , \end{aligned} \end{aligned}$$

the nonlinear operators are as follows

20 $${\mathfrak{N}}_{F}  = \varsigma _{3} \frac{{\partial ^{3} F(\eta ,\ddot{p})}}{{\partial \eta ^{3} }} - \left( {\frac{{\partial F(\eta ,\ddot{p})}}{{\partial \eta }}} \right)^{2}  + 2F(\eta ,\ddot{p})\left( {\frac{{\partial ^{2} F(\eta ,\ddot{p})}}{{\partial \eta ^{2} }}} \right) + \frac{{\varsigma _{1} }}{{\varsigma _{2} }}M\frac{{\partial F(\eta ,\ddot{p})}}{{\partial \eta }} - {\mathbb{U}}\left( {\frac{{\partial F(\eta ,\ddot{p})}}{{\partial \eta }} + \frac{\eta }{2}\frac{{\partial ^{2} F(\eta ,\ddot{p})}}{{\partial \eta ^{2} }}} \right) - G(\eta ,\ddot{p})^{2} ,{\mathfrak{N}}_{G}  = \varsigma _{2} \varsigma _{3} \frac{{\partial ^{2} G(\eta ,\ddot{p})}}{{\partial \eta ^{2} }} - \varsigma _{2} {\mathbb{U}}\left( {G(\eta ,\ddot{p}) + \frac{\eta }{2}\frac{{\partial G(\eta ,\ddot{p})}}{{\partial \eta }}} \right) - \varsigma _{1} MG(\eta ,\ddot{p}) - 2\varsigma _{2} (G(\eta ,\ddot{p})\frac{{\partial F(\eta ,\ddot{p})}}{{\partial \eta }} - F(\eta ,\ddot{p})\frac{{\partial G(\eta ,\ddot{p})}}{{\partial \eta }}),{\mathfrak{N}}_{\theta }  = \varsigma _{4} \frac{{\partial ^{2} \theta (\eta ,\ddot{p})}}{{\partial \eta ^{2} }} - Pr\varsigma _{5} ({\mathbb{U}}\eta  - 2F(\eta ,\ddot{p}))\frac{{\partial \theta (\eta ,\ddot{p})}}{{\partial \eta }},{\mathfrak{N}}_{\phi }  = \varsigma _{6} \frac{{\partial ^{2} \phi (\eta ,\ddot{p})}}{{\partial \eta ^{2} }} - {\mathcal{S}}_{c} \left( {{\mathbb{U}}\eta  + \kappa _{t} (1 + \gamma _{1} \theta (\eta ,\ddot{p}))^{s} \exp \left[ {\frac{{ - {\mathbb{E}}_{t} }}{{1 + \gamma _{1} \theta (\eta ,\ddot{p})}}} \right]} \right)\phi  + 2{\mathcal{S}}_{c} F(\eta ,\ddot{p})\frac{{\partial \phi (\eta ,\ddot{p})}}{{\partial \eta }}, $$

and we write the mth order deformation equation below

21 $$\begin{aligned} \begin{aligned} {\mathfrak {L}}_{F}\left[ F_{m}(\eta )-\xi _{m}F_{m-1}(\eta )\right] -\hbar _{F}\Re _{F}^{m}(\eta )=0,\\ {\mathfrak {L}}_{G}\left[ G_{m}(\eta )-\xi _{m}G_{m-1}(\eta )\right] -\hbar _{G}\Re _{G}^{m}(\eta )=0,\\ {\mathfrak {L}}_{\theta }\left[ \theta _{m}(\eta )-\xi _{m}\theta _{m-1}(\eta )\right] -\hbar _{\theta }\Re _{\theta }^{m}(\eta )=0,\\ {\mathfrak {L}}_{\phi }\left[ \phi _{m}(\eta )-\xi _{m}\phi _{m-1}(\eta )\right] -\hbar _{\phi }\Re _{\phi }^{m}(\eta )=0, \end{aligned} \end{aligned}$$

subject to the boundary conditions

22 $$\begin{aligned} \begin{aligned}{}&F'(0)=0,\quad F(0)=0,\quad G(0)=0,\quad \theta '(0)+{\mathfrak {B}}_{i1}(1-\theta (0))=0,\quad \phi (0)=0,\\&F'(1)=,\quad G(1)=0,\quad F(1)=0,\quad \theta '(1)-{\mathfrak {B}}_{i2}\theta (1)=0,\quad \phi (1)=0, \end{aligned} \end{aligned}$$

here

23 $$\begin{aligned} \begin{aligned} \Re _{F}^{m}(\eta )=&\varsigma _{3}F'''_{m-1}-\sum _{i=0}^{k-1}F'_{i}F'_{k-1-i}+2\sum _{i=0}^{k-1}F_{i}F''_{k-1-i}+\frac{\varsigma _{1}}{\varsigma _{2}}MF'_{m-1}-{\mathbb {U}}\left( F'_{m-1}+\frac{\eta }{2}F''_{m-1}\right) -\sum _{i=0}^{k-1}G_{i}G_{k-1-i},\\ \Re _{G}^{m}(\eta )=&\varsigma _{2}\varsigma _{3}G''_{m-1}-\varsigma _{2}{\mathbb {U}}\left( G_{m-1}+\frac{\eta }{2}G'_{m-1}\right) -\varsigma _{1}MG_{m-1}-2\varsigma _{2}\left( \sum _{i=0}^{k-1}G_{i}F'_{k-1-i}-\sum _{i=0}^{k-1}F_{i}G'_{k-1-i}\right) ,\\ \Re _{\theta }^{m}(\eta )=&\varsigma _{4}\theta ''_{m-1}-Pr\varsigma _{5}({\mathbb {U}}\eta \theta '_{m-1})-2\sum _{i=0}^{k-1}F_{i}\theta '_{k-1-i},\\ \Re _{\phi }^{m}(\eta )=&\varsigma _{6}\phi ''_{m-1}-{\mathcal {S}}_{c}\left( {\mathbb {U}}\eta +\kappa _{t}(1+\gamma _{1}\theta _{m-1})^{s}\exp \left[ \frac{-{\mathbb {E}}_{t}}{1+\gamma _{1}\theta _{m-1}}\right] \right) \phi _{m-1}+2{\mathcal {S}}_{c}\sum _{i=0}^{k-1}F_{i}\phi '_{k-1-i}, \end{aligned} \end{aligned}$$

and $$\xi _{m}={\left\{ \begin{array}{ll} 1,\quad m>1\\ 0,\quad m\le 1. \end{array}\right. }$$

 The final form of series solution obtained as a result is as follows

24 $$\begin{aligned} \begin{aligned} F_{m}(\eta )=F_{m}^{*}(\eta )+F_{0}(\eta ),\\ G_{m}(\eta )=G_{m}^{*}(\eta )+G_{0}(\eta ),\\ \theta _{m}(\eta )=\theta _{m}^{*}(\eta )+\theta _{0}(\eta ),\\ \phi _{m}(\eta )=\phi _{m}^{*}(\eta )+\phi _{0}(\eta ), \end{aligned} \end{aligned}$$

the special functions $F_{m}^{*}(\eta ),\,G_{m}^{*}(\eta ),\,\theta _{m}^{*}(\eta )\text { and }\phi _{m}^{*}(\eta )$ are computed trough Eq. (21) and general series form solutions are obtained as a result.

### Convergence analysis

**Figure 2:**
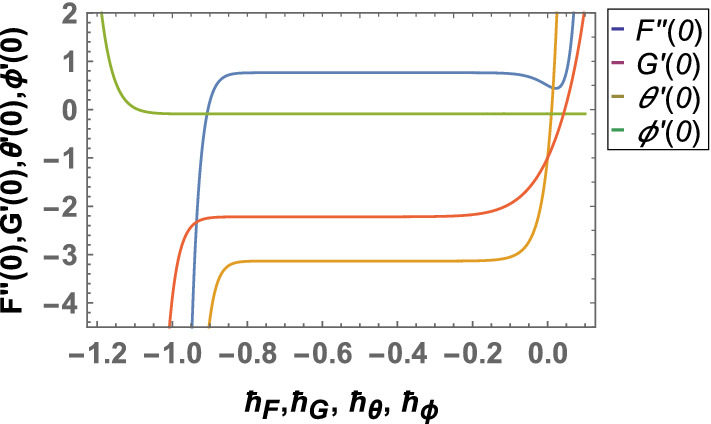
Combined h-curves.

After computing the series form solution through homotopic approach the convergence of results is optimized through $\hbar$-plots. The $\hbar$ curves are plotted for velocity, temperature and concentration profile at $23{rd}$ order in Fig. 2. It is observed that convergent regions of $\hbar$ are $-0.85<\hbar _{F}<-0.2$, $-0.9<\hbar _{G}<-0.3$, $-0.8<\hbar _{\theta }<-0.15$ and $-1.1<\hbar _{\phi }<-0.1$. Furthermore, series solution for velocity temperature and concentration are illustrated in Table 4 after fixing values of all fluid parameters. It is noted that the convergent series solutions are obtained at $33{rd}$, $38{th}$ and $41{st}$ iteration correct up to 6 decimal places.

**Table 4:** Series solution at various orders of approximations when $M = 3.3,\,{\mathbb {U}} = 1.2,\, Pr = 6.7,\,\gamma _{1} = 0.49,\,\kappa _{t} = 0.1,\,{\mathcal {S}}_{c} = 1.9,\,{\mathbb {E}}{t} = 0.6,\, \alpha _{1} = 1.7,\,\alpha _{2} = 2.2,\,{\mathfrak {B}}_{i1} = 0.09,\,{\mathfrak {B}}_{i2} = 2.6,\, s = 1.5.$

Order of approx.	$-F(\eta )$	$-G(\eta )$	$-\theta (\eta )$	$-\phi (\eta )$
7	0.429023	3.5656	0.0852444	2.23195
10	0.437133	3.58399	0.0855635	2.32442
12	0.438413	3.58679	0.085835	2.34994
15	0.438943	3.58791	0.0862952	2.36694
21	0.43907	3.58817	0.0872769	2.37705
24	0.439074	3.58818	0.0872941	2.37705
33	0.439075	3.58818	0.0872941	2.37995
38	0.439075	3.58818	0.0879241	2.37995
41	0.439075	3.58818	0.0879241	2.37995

## Results and discussion

The flow problem is simulated in the fluid domain to depict the behavior of hybrid nanofluid under various effect and physical parameters. In this regard, each fluid phenomena is discussed in detail for hybrid nanofluid in following sections for velocity, temperature and concentration profile.

### Magnetic interaction parameter

**Figure 3:**
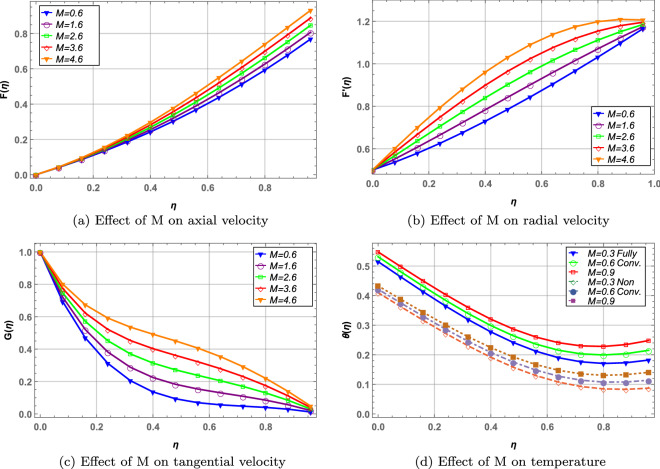
Effect of magnetic interaction parameter on velocity and temperature profile.

The ratio of electromagnetic force to the viscous forces in a fluid flow is characterized by magnetic interaction parameter, *M*. In Fig. 3 behavior of radial, axial, tangential velocities and temperature is plotted against increasing values of *M*. As *M* increases, the viscous forces in fluid layers decreases causing increase in velocity in all directions. This increase in velocity results in increased temperature of hybrid nanofluid. The temperature profile is analyzed for both fully convective and non-convective boundaries. It is observed that temperature in fully convective boundaries is higher when compared with thermally non-convective (${\mathfrak {B}}_{i1}=\infty ,\, {\mathfrak {B}}_{i2}=0$) rotating disks. In case of convective boundaries the disks are exposed to the heat transfer through convection which is further governed by Newton’s law of cooling, causing elevated temperature of the hybrid nanofluid.

### Unsteadiness parameter

**Figure 4:**
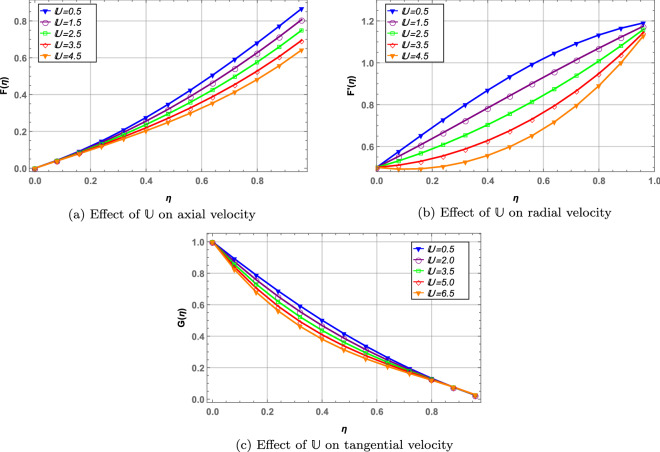
Effect of unsteady parameter on velocity profile.

**Figure 5:**
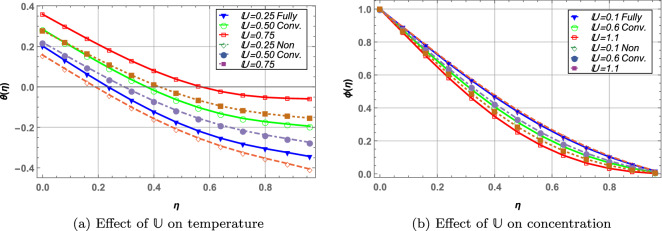
Effect of unsteady parameter (fully convective vs. non-convective) on temperature and concentration profile.

Unsteadiness parameter, ${\mathbb {U}}$ is inversely related to rotation of the disks. In Fig. 4 axial, tangential and radial velocities decrease with increase in ${\mathbb {U}}$. As the parameter ${\mathbb {U}}$ increases this causes a decrease in rotation of disks resulting in lower velocity profile of the hybrid nanofluid. In Fig. 5, temperature of the fluid increases while concentration decreases against higher values of ${\mathbb {U}}$. It is also noted that fully convective boundary case depicts elevated temperature and slightly lower concentration of the fluid when compared with non-convective boundary case.

### Stretching parameters

**Figure 6:**
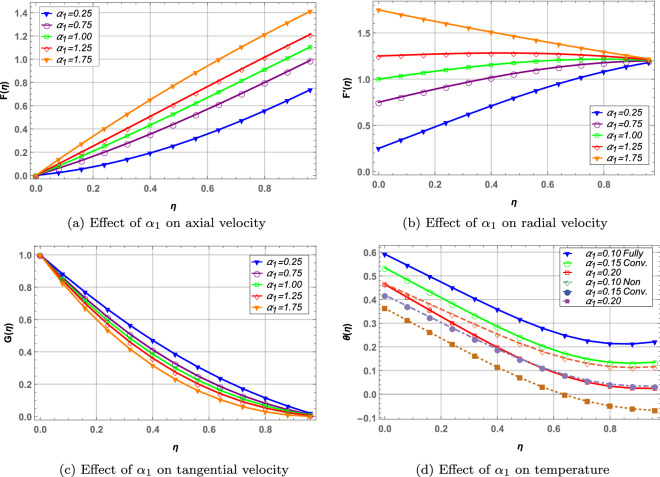
Effect of right disk stretching parameter on velocity and temperature profile.

**Figure 7:**
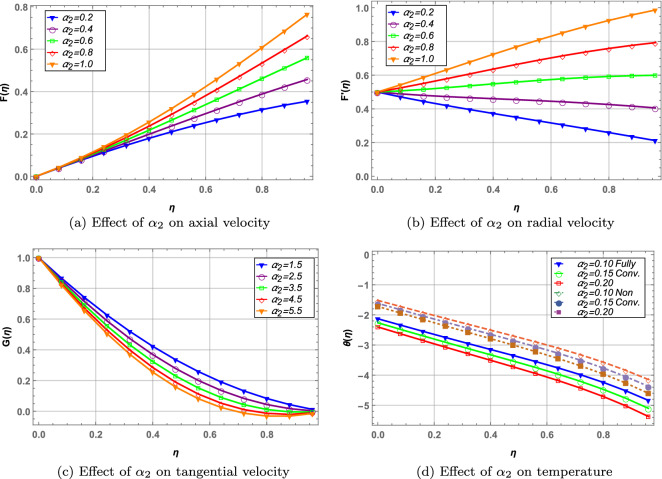
Effect of unsteady parameter on velocity, temperature and concentration profile.

The parameters $\alpha _{1}$ and $\alpha _{2}$ are the ratio of stretching rate to rotation of the disks. $\alpha _{1}$ and $\alpha _{2}$ increases the radial and axial velocity whereas the tangential velocity decreases in Figs. 6, 7. Higher values of stretching parameter results from elevated stretching rate and decreased disks rotation. As a result, velocity in tangential direction direction decreases due to drop in the rotation of disks. Temperature of the hybrid nanofluid in Figs. 6(d), 7(d) decreases with increase in both $\alpha _{1}$ and $\alpha _{2}$. Moreover, the fully convective boundary case results in higher temperature when $\alpha _{1}$ is increased whereas contrary is observed in case of $\alpha _{2}$.

### Volume fraction

**Figure 8:**
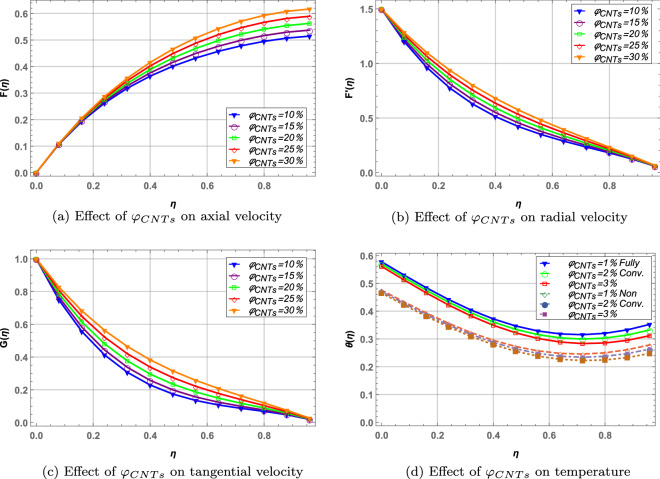
Effect of MWCNTs volume fraction on velocity and temperature profile when $\varphi _{UO_{2}}=2\%$.

In Fig. 8, volume fraction of $UO_{2}$ is kept constant and $\varphi _{CNTs}$ is increased. Increasing the concentration of *MWCNTs* in blood increases the velocity of the hybrid nanofluid in radial, tangential and axial direction. Temperature of the hybrid nanofluid decreases with increased volume fraction of *MWCNTs*. It is also observed that fully convective boundaries offer higher temperature than non-convective boundaries in case of increasing volume fraction of carbon nanotubes.

### Prandtl number and chemical reaction

**Figure 9:**
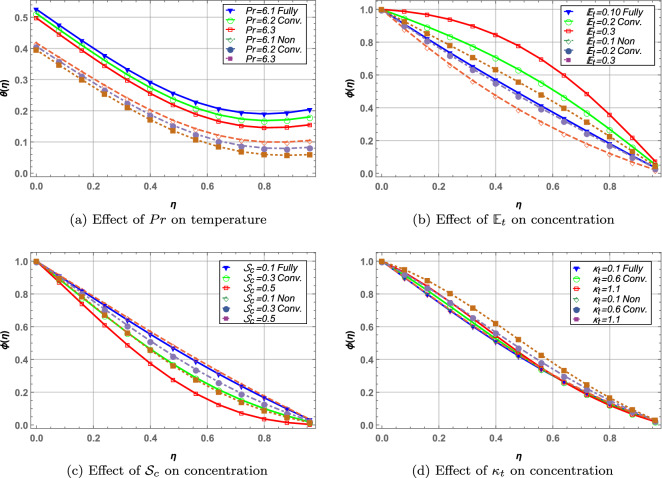
Effect of Prandtl number, activation energy parameter, Schmidt number and chemical reaction parameter on temperature and concentration profile.

Convective and non-convective boundary cases for temperature and concentration are shown in Fig. 9. Increase in Prandtl number *Pr* decrease fluid temperature due to reduced thermal conductivity inside the fluid. Fully convective boundaries offer higher temperature as compared to non-convective disks when *Pr* is increased. Increase in activation energy parameter ${\mathbb {E}}_{t}$ and chemical reaction $\kappa _{t}$ elevates the fluid concentration whereas Schmidt number ${\mathcal {S}}_{c}$ shows opposite results. Fully convective disks result in higher concentration of fluid in comparison with non-convective boundaries in case of increasing ${\mathbb {E}}_{t}$ and contrary behavior is observed in case of ${\mathcal {S}}_{c}$ and $\kappa _{t}$.

### Skin friction

**Figure 10:**
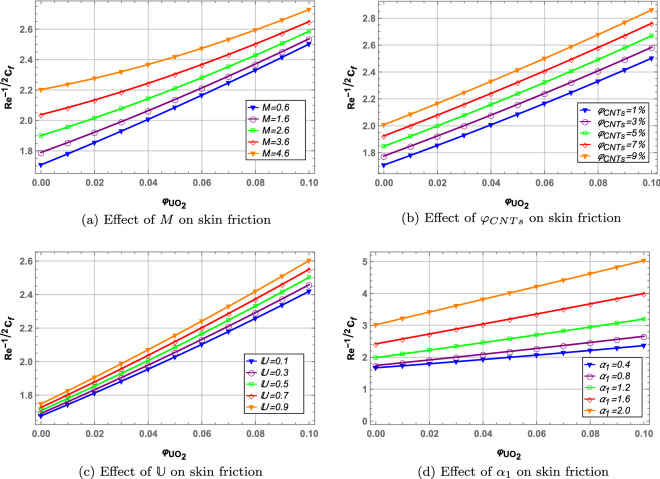
Skin friction profile.

Figure 10 depict the skin friction profile of hybrid nanofluid with $\varphi _{UO_{2}}$ on the x-axis. Increase in magnetic interaction parameter *M* increases the skin friction due to enhanced Lorentz forces between fluid particles. Increasing volume fraction of *CNTs* increases the skin friction as nore solid nanoparticles move through the fluid. Unsteady parameter *U* and stretching parameter $\alpha _{1}$ also increases the fluid skin friction. Furthermore, it is noted that as volume fraction of $UO_{2}$ increases on the x-axis, the skin friction increases.

### Heat transfer rate

**Figure 11:**
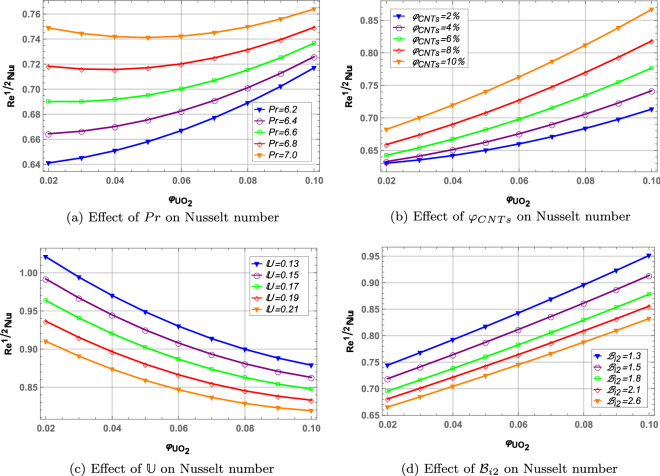
Heat transfer profile.

Rate of heat transfer is the ratio of convective heat transfer and conductive heat transfer during a fluid flow. Figure 11 presents the rate of heat transfer against increasing values of *Pr*, $\varphi _{CNTs}$, ${\mathbb {U}}$ and ${\mathfrak {B}}_{i}$. Increase in Prandtl number increases the kinematic viscosity of the fluid resulting in more heat transfer rate throughout the fluid. Increase in volume fraction of *CNTs* increases the heat transfer in Fig. 11(b) as increased nanoparticles of carbon nanotubes offer higher thermal conductivity. Unsteady parameter and Biot number decrease the heat transfer rate in Figs. 11(c,d). Furthermore, Nusselt number increases along x-axis with increase in $\varphi _{UO_{2}}$ in case of *Pr*, $\varphi _{CNTs}$ and ${\mathfrak {B}}_{i}$ while a decrease in heat transfer with increasing volume fraction of $UO_{2}$ in case of ${\mathbb {U}}$.

### Mass transfer rate

**Figure 12:**
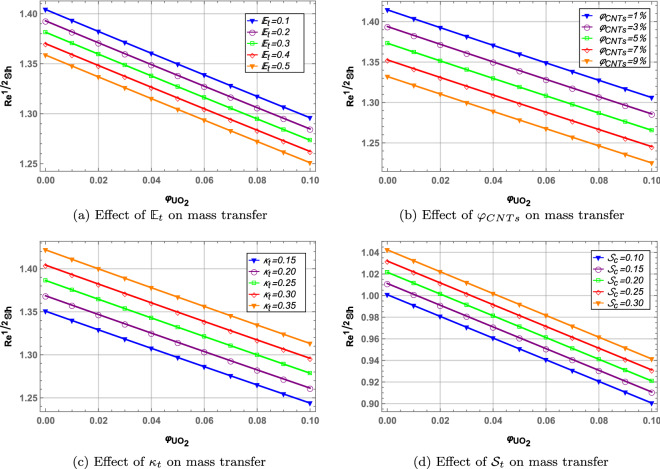
Mass transfer profile.

The ratio of mass transfer by convection and diffusion is the Sherwood number which is the mass transfer rate in the fluid. Figure 12 presents the behavior of Sherwood number with increasing values of activation energy parameter ${\mathbb {E}}_{t}$, *CNTs* volume fraction $\varphi _{CNTs}$, chemical reaction parameter $\kappa _{t}$ and Schmidt number ${\mathcal {S}}_c$. ${\mathbb {E}}_{t}$ and $\varphi _{CNTs}$ decreases the mass transfer rate through the hybrid nanofluid. $\kappa _{t}$ and ${\mathcal {S}}_c$ increases the mass transfer through the blood hybrid nanofluid. Moreover, $\varphi _{UO_{2}}$ decreases the mass transfer in all cases due to high density of the $UO_{2}$ nanoparticles.

## Conclusion

Current investigation focuses on simulating an unsteady and convective flow of blood based hybrid nanofluid undergoing chemical reaction with activation energy. A novel semi-analytical approach that is homotopy analysis method is utilized to solve the modeled system of non-linear ODEs. Convergence control parameters $\hbar _{i}$ are plotted for velocity, temperature and concentration equation presenting the convergence region of $\hbar$. Moreover, convergent series solutions are also computed at $33{rd}$, $38{th}$ and $41{st}$ iterations in tabular form. Velocity (axial, radial and tangential), temperature, concentration, skin friction, heat and mass transfer of the fluid are simulated in the entire domain and physical interpretations are drawn. Graphical analysis reveals the following major outcomes of this study:

- Radial, tangential and axial velocity of the hybrid nanofluid increases with increase in *M* and $\varphi _{CNTs}$ while opposite behavior is observed in case of increasing values of ${\mathbb {U}}$ .
- Increase in $\alpha _{1}$ and $\alpha _{2}$ elevates the radial and axial velocities whereas tangential velocity decreases.
- Temperature of the hybrid nanofluid boosts with higher values of *M* and ${\mathbb {U}}$ while contrasting results are observed in case of $\alpha _{i}$, $\varphi _{CNTs}$ and *Pr*.
- Convective boundary conditions result in higher temperature of hybrid nanofluid when compared with non-convective boundary condition case.
- Increase in ${\mathbb {U}}$ and ${\mathcal {S}}_{c}$ decreases concentration of hybrid nanofluid while an increase is observed in case of ${\mathbb {E}}_{t}$ and $\kappa _{t}$.
- Skin friction increases with increase in both volume fractions $\varphi _{UO_{2}}$ and $\varphi _{CNTs}$.
- The rate of heat transfer decreases with increasing volume fraction $\varphi _{UO_{2}}$ in case of higher values of ${\mathbb {U}}$.
- Increase in volume fractions of both nanoparticles $UO_{2}$ and *CNTs* decreases the rate of mass transfer in the hybrid nanofluid.

## Data Availability

All data generated and analyzed during this study are included in this article with its reference list.
